# Divergent immunohistochemical expression of CD21 and CD23 by follicular dendritic cells with increasing grade of follicular lymphoma

**DOI:** 10.1186/s12957-019-1659-8

**Published:** 2019-07-03

**Authors:** Fisnik Kurshumliu, Fatlinda Sadiku-Zehri, Ardita Qerimi, Zana Vela, Fisnik Jashari, Samir Bytyci, Vlore Rashiti, Shemsedin Sadiku

**Affiliations:** 1 Institute of Anatomic Pathology, University Clinical Center/Faculty of Medicine, University of Pristina, Pristina, Kosovo; 2 Institute of Histology, University Clinical Center/Faculty of Medicine, University of Pristina, Pristina, Kosovo; 3 Institute of Anatomic Pathology, University Clinical Center of Pristina, Pristina, Kosovo; 4 Hematology Clinic, University Clinical Center/Faculty of Medicine, University of Pristina, Rr.Bulevardi i Dëshmorëve, 10000 Pristina, PN Kosovo

**Keywords:** CD21, CD23, Immunohistochemistry, Follicular lymphoma

## Abstract

**Background:**

Ultrastructural and immunohistochemical differences have been described in FDCs of primary and secondary follicles, illustrating the highly compartmentalized structure of lymph follicles. Differences in FDC immunophenotype in different grades of FL may reflect some parallelism between reactive and neoplastic conditions in terms of FDC-B cell interaction and may be used as a valuable additional tool for grading FL.

**Methods:**

A total of 60 paraffin blocks from patients with follicular lymphoma, 30 cases each of grade 1 and 3, were retrieved from our archive. Immunohistochemical analysis was carried out for CD21, CD23, cyclin A, and Ki-67.

**Results:**

Our study demonstrates that during evaluation, six patterns of FDC distribution were distinguished. The intensity of stain for CD21 was not statistically significant in grade 1 and grade 3 FL (*p* = 0.340). In contrast, grade 3 FLs exhibited a significant decrease of CD23 expression by the FDCs (*p* < 0.001). By CD21 stain, there was no significant difference in the distribution of pattern 1 in grades 1 and 3 (*p* = 0.098). In contrast, in grade 3, this pattern was significantly less observed by CD23 stain (*p* = 0.016). The same was observed for pattern 2 for CD21 (*p* = 0.940) and CD23 (*p* = 0.010) and pattern 4 for CD21 (*p* = 0.305) and CD23 (*p* = 0.005), respectively. Distribution of pattern 5 was significantly different between grades 1 and 3 both for CD21 (*p* = 0.005) and CD23 (*p* < 0.001). Distribution of patterns 2 and 6 was not significantly different between grades 1 and 3 for CD21 and CD23. The values of cyclin A and Mib-1 were also significantly different between grades 1 and 3 (*p* < 0.001).

**Conclusions:**

The observed patterns of FDCs lead us to believe that similar to reactive lymph node follicles, neoplastic follicles in FL, at least in early stages, have an organized structure. Hypothetically, with CD21, CD23, and cyclin A immunohistochemistry, the sequence of events in FL progression may be traced.

## Introduction

Immunohistochemical identification of FDC networks and demonstration of germinal center origin of tumor cells are key diagnostic features in follicular lymphoma (FL) [1]. Three-tiered histological grading of FL, based on quantification of neoplastic centrocytes and centroblasts in the neoplastic follicles, is hampered by intra- and interobserver variability [1, 2]. Some morphologically low-grade FLs have a high proliferative index and a more aggressive behavior [1]. Generally, neoplastic follicles in FL, in contrast to reactive germinal centers, have a lower proliferative index as measured by Ki-67 immunohistochemistry. However, with the increase of FL grade, the Ki-67 index also increases [3, 4]. Given that Ki-67 marks the cells throughout the cell cycle, determination of proliferation index in follicular lymphoma by this antibody may suffer certain interpretation issues due to the overlapping of signal. On the other hand, cyclin A, a member of the cyclin family, which binds to S phase CDk2 is more restrictive to the S phase of the cell cycle, hence staining fewer cells. Accordingly, it gives lesser overall values but a better discrimination also in terms of the distribution of proliferating cells [5]. Despite histological grading and determination of Follicular Lymphoma International Prognostic Index (FLIPI), the evolution of FL is heterogeneous [1, 6–8]. Some patients present with indolent disease undergoing several relapses while others follow a more aggressive course [8].

Gene expression profile studies have shown that the prognosis of FL is also influenced by the type, number, and activation of cells in the microenvironment [6–8]. It is hypothesized that the number and distribution of non-malignant cells can in part serve as surrogate markers for clinically relevant gene expression signatures [6].

Follicular dendritic cells (FDCs) are a unique type of cell characterized by localization within lymphoid follicles, intricately entangled cytoplasmic processes, the ability to trap and retain immune complexes, and expression of molecules involved in the proliferation and differentiation of B cells [9]. There are a number of antibodies suited for formalin-fixed paraffin-embedded tissue, by which FDCs in both reactive and neoplastic follicles are recognized. Some of the markers more frequently employed are antibodies to complement receptors such as CD21 and CD35, IgE FC receptors such as CD23, and IgG FC receptors such as CD32 [9]. Ultrastructural and immunohistochemical differences have been described in FDCs of primary and secondary follicles, illustrating the highly compartmentalized structure of lymph follicles [9]. In reactive lymph nodes, FDCs of both primary and secondary follicles are recognized by CD21 and CD35. FDCs in germinal center (GC) light zones (LZ) additionally upregulate CD23 and CD32 [9]. Up- and downregulation of CD23 by the FDCs seems to be related to centrocytic and centroblastic differentiation of the B cells, respectively. Hence, differences in FDC immunophenotype in different grades of FL may reflect some parallelism between reactive and neoplastic conditions in terms of FDC-B cell interaction and may be used as a valuable additional tool for grading FL.

## Methods

**Fig. 1:**
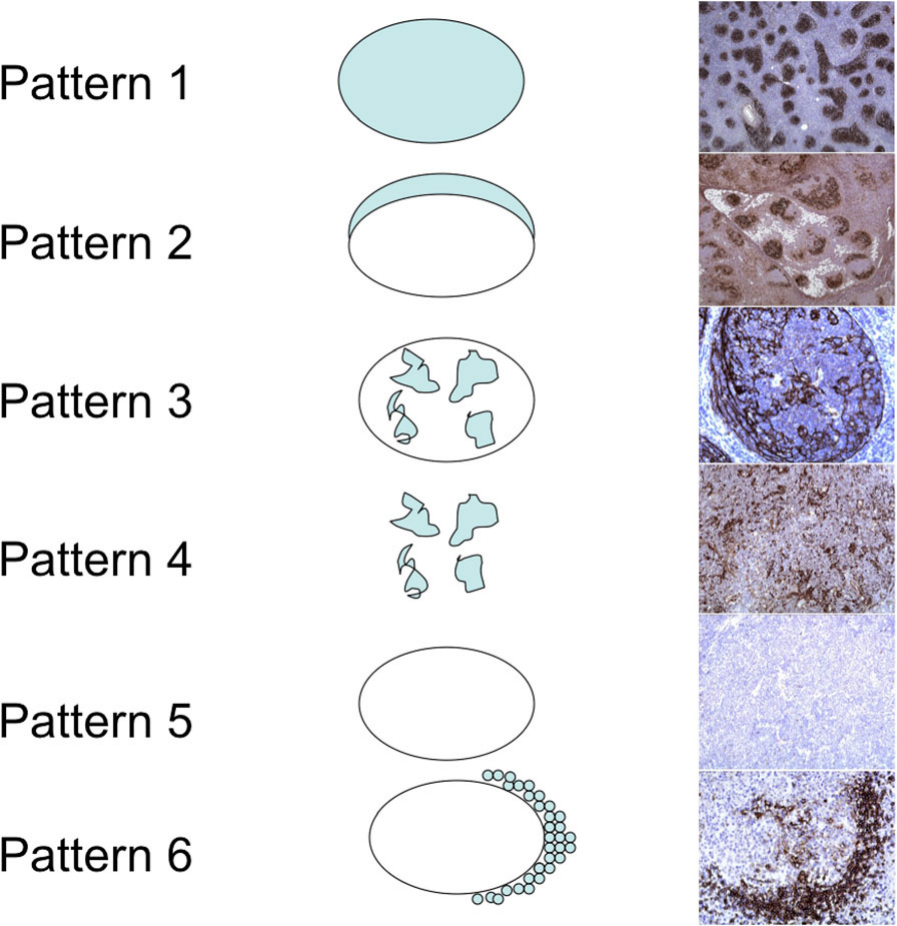
Patterns of CD21 and CD23 expression by the follicular dendritic cells. Pattern 1 describes diffuse staining of FDCs in the germinal center while pattern 2 demonstrates the peripheral staining in a “crescetic” fashion. Pattern 3 shows broken networks of the FDCs while the follicular outline is preserved. In pattern 4, the follicular outline is lost. Pattern 5 shows lack of stain and, in pattern 6, there is staining of FDC extensions around the individual mantle zone B cells

A total of 60 paraffin blocks from patients with follicular lymphoma, 30 cases each of grade 1 and 3, were retrieved from our archive. Biopsy samples had been fixed in 10% neutral buffered formalin and sectioned in 3–4micron sections. Immunohistochemical analysis was carried out for CD21, CD23, cyclin A, and Ki-67 (Table 1). Antigens were retrieved by placing the slides in a target retrieval solution for 45 min at 95–98 °C. The slides were incubated with the primary antibody for 30 min. The visualization was carried out with dextran polymer conjugated with peroxidase and secondary antibody (EnVision+, DAKO, Denmark, K534011) for 30 min. Interpretation of CD21 and CD23 stain was carried out by focusing on the follicular dendritic cell network in the germinal centers and the slender extensions of FDCs in the mantle zone. Staining of lymphocytes in the mantle and interfollicular zone was not taken into account. The intensity of staining was score-ranked from 1 through 5. During evaluation, six patterns of FDC distribution were distinguished (Fig. 1). Pattern 1 was complete staining of FDC networks throughout the neoplastic follicle. Pattern 2 was peripheral staining of the neoplastic follicle in a “crescentic” fashion, resembling light zone of the reactive germinal center. In pattern 3, the FDC network was broken throughout the follicle while the follicular outline was preserved. This pattern is similar to what is observed in the progressively transformed germinal center. The follicular outline was missing in the pattern 4. Pattern 5 consisted of complete lack of visualization of the FDC network. Pattern 6 highlighted FDC extensions in the mantle zone. All the patterns present in an individual lymph node were registered. SPSS was employed for statistical analysis.

**Table 1:** Antibodies, clones, and pretreatment

Antibody	Clone	Source	Dilution	Pretreatment
CD21	1F8	DAKO	1:20	pH 6.0
CD23	SP23	Labvision	1:100	PH9.0
Cyclin A	NCL-CYCLIN A	Novocastra	1:50	pH 9.0
Ki-67	MIB-1	DAKO	1:100	pH 9.0

## Results

A total of 60 cases of nodal follicular lymphoma were analyzed by immunohistochemistry for expression of CD21 and CD23 by FDCs, and cyclin A, and Ki-67 by the tumor cells (Table 1). The study comprised thirty cases each of grade1 and 3 FL, respectively.

The intensity of stain for CD21 was not statistically significant in grade 1 and grade 3 FL (*p* = 0.340). In contrast, grade 3 FLs exhibited a significant decrease of CD23 expression by the FDCs (*p* < 0.001). We further analyzed the distribution of FDC patterns as marked by CD21 and CD23. Statistical analysis revealed that by CD21 stain, there was no significant difference in the distribution of pattern 1 in grades 1 and 3 (*p* = 0.098). In contrast, in grade 3, this pattern was significantly less observed by CD23 stain (*p* = 0.016). The same was observed for pattern 2 for CD21 (*p* = 0.940) and CD23 (*p* = 0.010) and pattern 4 for CD21 (*p* = 0.305) and CD23 (*p* = 0.005), respectively. Distribution of pattern 5 was significantly different between grades 1 and 3 both for CD21 (*p* = 0.005) and CD23 (*p* < 0.001). Distribution of patterns 2 and 6 was not significantly different between grades 1 and 3 for CD21 and CD23. The values of cyclin A and Mib-1 were also significantly different between grades 1 and 3 (*p* < 0.001).

## Discussion

The majority of B cell lymphomas originate from the GC. The preferential localization of lymphoma cells in the GC suggests a unique relationship between tumor cells and their microenvironment [6, 9–19]. The generation and transformation of lymphomas occurs in close association with FDCs [18, 20]. FDCs contribute to lymphomagenesis by preventing apoptosis as well as by promoting the proliferation of transformed B cells. Lymphoma cells emerge by the acquisition of additional genetic changes or through adaptation to the protumorigenic environment provided by the signaling molecules of the FDCs [18, 20–22]. FDCs are not homogenous but are composed of different subpopulations, as defined by their surface markers. For survival, different types of lymphoma cells may require unique FDC subsets providing different surface molecules [6, 9–19]. Alternatively, distinct types of lymphomas may influence the differentiation of FDCs into different subpopulations [12, 18, 20].

The cellular microenvironment in FL is of biological and clinical importance [2, 6–8, 14, 15, 20, 21, 23–26]. It is hypothesized that the frequency and distribution of non-malignant cells in the microenvironment can in part serve as surrogate markers for the clinically relevant gene expression signatures. This issue has been the focus of numerous immunohistochemical studies which evaluated T cell subsets, macrophages, mast cells, microvessel density, and FDCs [6–8, 10, 12, 19, 21, 23–25, 27–29].

Ultrastructural differences have been described between FDCs in primary and secondary follicles, also within the light zone (LZ) and dark zone (DZ) of GC [9, 13, 17, 30, 31]. A series of articles describe the differences in CD21 and CD23 expression during GC development [9, 17, 20, 31–33]. Antibodies to the complement receptors, CD21 and CD35, recognize FDCs in both primary and secondary follicles whereas FDCs in GC LZ upregulate two Fc receptors namely, CD23 and CD32 [9, 31]. In fact, at the outset, we had tested the following antibodies: CD21, CD23, CD35, and CNA42 in terms of the following: (1) intensity of stain, (2) distribution of positive FDCs, and (3) percentage of positive follicles. CD21 was the antibody that gave the best stains and second to it was CD23. By using these two, we could cover the complement receptor and IgE FC receptor group of FDC antibodies. Additionally, we consider that the utility of CD23 is not only in FDC identification but also in lymphoma diagnosis and classification since it is a well-established marker in routine immunohistochemistry panels in lymphoma diagnosis and classification. Hence, we used the two former mentioned antibodies in the subsequent steps of our study. It is well known that centrocytes populate GC LZ and centroblasts populate GC DZ. When these facts are observed from follicular lymphoma standpoint, it may well be that in grade 1 FL, where neoplastic follicles are mainly composed of centrocytes, the FDC network resembles that of the primary follicle or GC LZ, hence expressing both CD21 and CD23. In contrast, in grade 3 FL, neoplastic follicles are mainly composed of centroblasts so the FDC network resembles that of the GC DZ, thereby expressing CD21 but losing CD23. This is supported by the findings of our study where we show that generally, as shown in the first two rows of Table 2, the distribution of CD21 was the same across categories of grade whereas this was not the case with CD23. Specifically, in FL grade 1, patterns 1, 2, 3, 4, and 6 were visualized by both CD21 and CD23 stain. In contrast, in FL grade 3, patterns 1, 2, and 4 of FDCs were lost by CD23. Distribution of pattern 5 was significantly different between grades 1 and 3 both for CD21 (*p* = 0.005) and CD23 (*p* < 0.001).

**Table 2:**
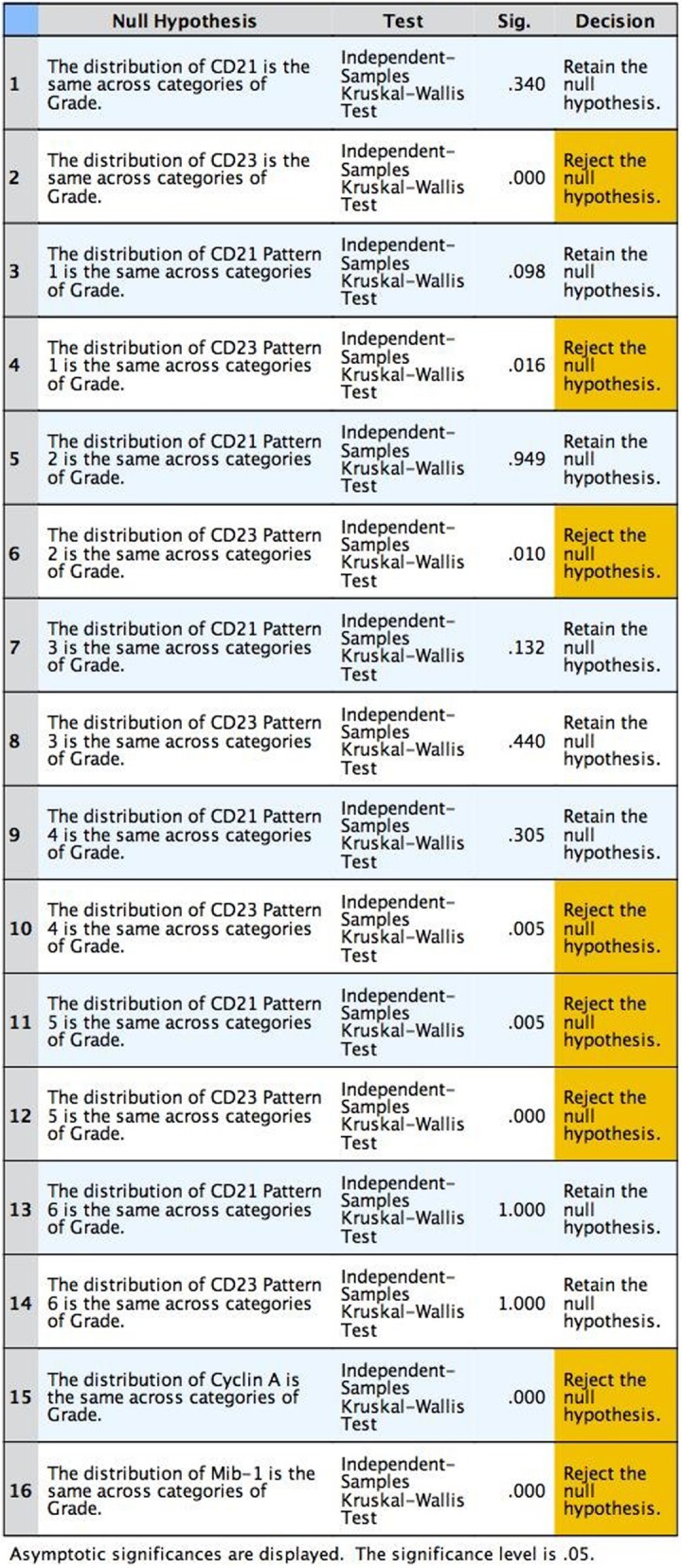
Hypothesis test Summary

Asymptotic significances are displayed. The significance level is .05

**Fig. 2:**
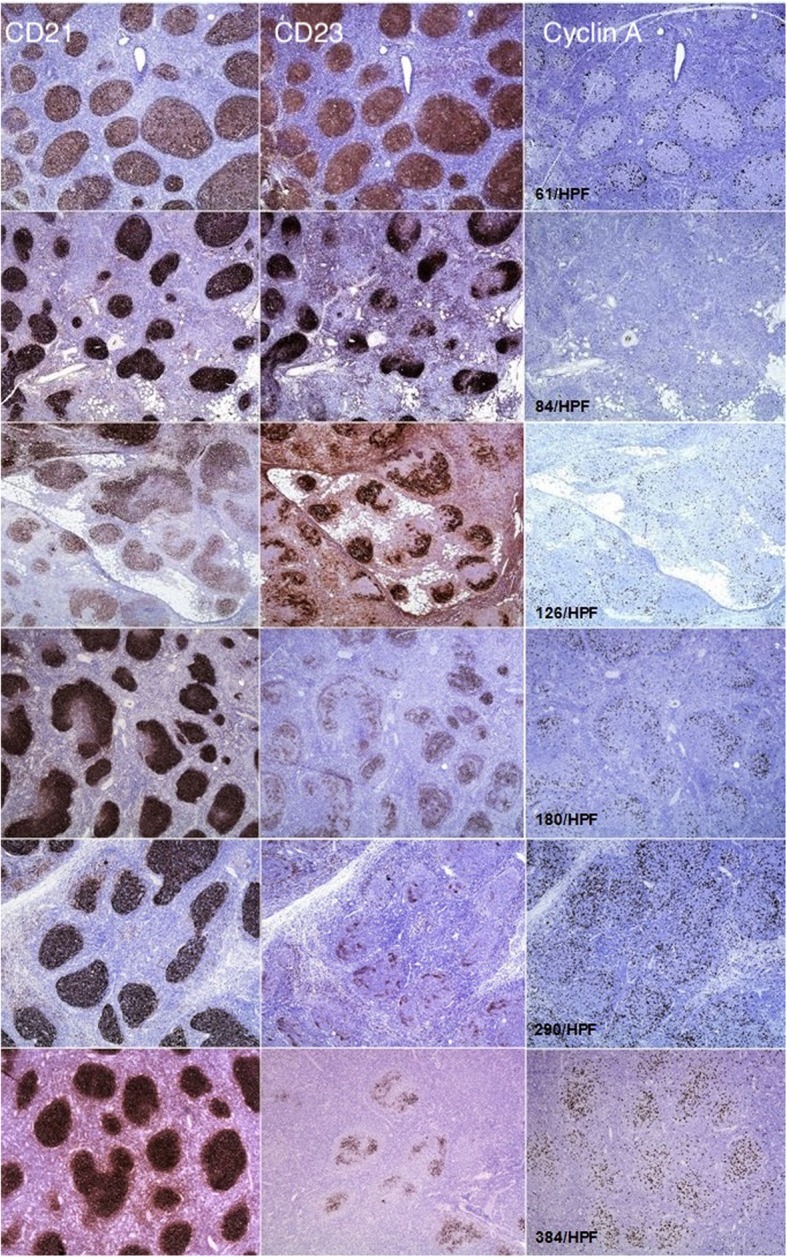
CD21 (first column), CD23 (second column), and cyclin A (third column) stain in grade 1 (upper three rows) and grade 3 (lower three rows) FL. The picture demonstrates increasing cyclin A values per high-power field (HPF) with downregulation of CD23

Hence, we show that in a typical grade 1 FL, the FDCs have the phenotype of GC LZ, staining intensely and completely for CD21 and CD23. In contrast in grade 3, the FDCs have the phenotype of GC DZ, with downregulation of CD23 (Fig. 2). We also demonstrate that these findings correlate with cyclin A and Ki-67 values (Fig. 2). Cases with intense and complete staining with CD21 and CD23, typically grade 1 FLs, demonstrate low values of cyclin A and Ki-67. In contrast, grade 3 FLs with loss of CD23 demonstrate increasing values of cyclin A and Ki-67. Concerning the issue of why we left out the grade 2 cases, we should emphasize that by the exclusion of grade 2 cases, we intended to analyze the “two ends of the spectrum.” As it is the case that histological classification of FL is hampered by inter- and intraobserver variability, we considered that most overlap is, in fact, in grade 2 FL. Hence, by analyzing the grade 1 at one end, and grade 3 at the other, the phenotypic changes of FDC should be more conspicuous and would give us a better idea of the whole process. Additionally, we consider that by utilizing this system, a significant proportion of grade 2 FLs would subsequently reclassify into either grade 1 or grade 3 FL.

Ki-67 generally gave higher values due to the fact that it detects cells in any phase of the mitotic cycle [34]. In contrast, cyclin A detects cells in S phase of the cell cycle [35] thereby giving generally lower values but important insights in terms of the distribution of proliferating cells. In our study, we observed that in cases with intense CD21/CD23 stain, a typical pattern for FL grade 1, the cyclin A-positive cells were situated at the periphery of the neoplastic follicle. In contrast, with CD23 loss, the cyclin A-positive cells acquired a more haphazard distribution. This was not observed with Ki-67 stain.

In conclusion, we observed similarities in FDC immunophenotype between reactive GCs and neoplastic follicles in FL. FDCs in grade 1 FL share a similar immunophenotype with FDCs in the primary follicle and GC LZ. Additionally, FDCs in grade 3 FL share a similar immunophenotype with FDCs in GC DZ. These findings are complemented with the proliferation rate of tumor cells as measured by cyclin A and Ki-67. Furthermore, the observed patterns of FDCs in correlation to the distribution of cyclin A-positive cells lead us to believe that similar to reactive follicles, neoplastic follicles in FL, at least in early stages, have an organized structure. Hypothetically, with CD21, CD23, and cyclin A immunohistochemistry, the sequence of events in FL progression may be traced. Further studies with a larger number of cases are necessary to provide more insights. Rigorous criteria in histological grading have to be employed for the selection of new cases to be tested.

## Data Availability

The data and material are at your disposition.
